# Melatonin improves the vitrification of sheep morulae by modulating transcriptome

**DOI:** 10.3389/fvets.2023.1212047

**Published:** 2023-10-18

**Authors:** Pengyun Ji, Yunjie Liu, Laiqing Yan, Yanquan Jia, Mengmeng Zhao, Dongying Lv, Yujun Yao, Wenkui Ma, Depeng Yin, Fenze Liu, Shuai Gao, Abulizi Wusiman, Kailun Yang, Lu Zhang, Guoshi Liu

**Affiliations:** ^1^College of Animal Science, Xinjiang Agricultural University, Urumqi, China; ^2^National Engineering Laboratory for Animal Breeding, College of Animal Science and Technology, China Agricultural University, Beijing, China; ^3^Beijing Jiaen Hospital, Beijing, China; ^4^Inner Mongolia Golden Grassland Ecological Technology Group Co., Ltd., Inner Mongolia, China

**Keywords:** embryo freezing, cryoprotectant, antioxidant, RNA-seq, DNA repair

## Abstract

Embryo vitrification technology is widely used in livestock production, but freezing injury has been a key factor hindering the efficiency of embryo production. There is an urgent need to further analyze the molecular mechanism of embryo damage by the vitrification process. In the study, morulae were collected from Hu sheep uterine horns after superovulation and sperm transfusion. Morulae were Cryotop vitrified and warmed. Nine morulae were in the vitrified control group (frozen), and seven morulae were vitrified and warmed with 10^−5^ M melatonin (melatonin). Eleven non-frozen morulae were used as controls (fresh). After warming, each embryo was sequenced separately for library construction and gene expression analysis. *p* < 0.05 was used to differentiate differentially expressed genes (DEG). The results showed that differentiated differentially expressed genes (DEG) in vitrified morulae were mainly enriched in protein kinase activity, adhesion processes, calcium signaling pathways and Wnt, PI3K/AKT, Ras, ErbB, and MAPK signaling pathways compared to controls. Importantly, melatonin treatment upregulated the expression of key pathways that increase the resistance of morulae against vitrification induced damage. These pathways include kinase activity pathway, ErbB, and PI3K/Akt signaling pathway. It is worth mentioning that melatonin upregulates the expression of XPA, which is a key transcription factor for DNA repair. In conclusion, vitrification affected the transcriptome of *in vivo*-derived Hu sheep morulae, and melatonin had a protective effect on the vitrification process. For the first time, the transcriptome profiles caused by vitrification and melatonin in sheep morulae were analyzed in single embryo level. These data obtained from the single embryo level provide an important molecular mechanism for further optimizing the cryopreservation of embryos or other cells.

## 1. Introduction

Cryopreservation is widely used for embryo preservation and has become routine of the commercial embryo transfer industry (1). Currently, there are two basic techniques being used for cell cryopreservation. i.e., the controlled slow-rate freezing and the vitrification. Compared to slow-rate freezing, vitrification cryopreservation does not require an expensive biological freezer and the method is simple and efficient. In addition, the vitrification does not produce ice crystals, but it possibly reduces the formation of ice crystals, and has fewer chilling associated injuries by increasing the temperature and transfer speed (2). Therefore, the survival rates of vitrification preserved ovine morulae and blastocysts derived both *in vivo* or *in vitro* were significantly higher than those of embryos cryopreserved with slow freezing techniques (*in vivo* embryo survival rate for slow freezing is 45.6% and that of vitrification is 67.1%. *In vitro* embryo survival rate for slow freezing is 7.3% and that of vitrification is 38.7%) (3–5). As a result, vitrification cryopreservation becomes the dominant method for preserving the domestic animal embryos. However, the process of vitrification can still cause damage to the embryo, resulting in embryo loss and abnormal fetal development. Mechanistically, the vitrification process causes some ultrastructural changes in embryo (6), which reduces the embryo’s quality by altering gene expression and increasing the level of apoptosis and damages mitogen-activated protein kinase (MAPK), an enzyme that is essential for embryonic development (7). It was suggested that the key to improve the efficiency of vitrification is to reduce the volume of the cryoprotectant and increase the cooling rate (8, 9).Thus, a very small amount (<0.01 mL) of vitrification solution was placed on the tip of a thin polypropylene band to preserve the embryos and immediately submerge them in liquid nitrogen (10). This modified vitrification process is referred to as Cryotop method. The small amount of vitrification medium around the embryos allows them to pass the critical temperature zone rapidly. Different vitrification processes affect the efficiency of cryopreservation. For example, Cryotop method preserved bovine oocytes had higher blastocyst rates compared to the traditional OPS (Open pulled straw) procedure (11). The cooling rate of Cryotop vitrification is similar to that of OPS vitrification, but the warming rate of Cryotop (approximately 48,000°C/min) is much higher than that of OPS (approximately 14,000°C/min) (12). This difference may explain the higher efficiency of the Cryotop method.

During the vitrification process, embryos that form a highly viscous glassy state behave like a solid, avoiding crystallization which causes embryonic damage (13), but the side effect of cryoprotectant can also cause embryonic injury (14). Thus, many studies have attempted to optimize the embryo vitrification process by adjusting the concentration of cryoprotective agents (CPAs) or the components of the vitrification solution (15, 16). One of the novel CPAs is melatonin. Melatonin (N-acetyl-5-methoxytryptophan, MT) is a derivative of tryptophan. It is a potent antioxidant and free radical scavenger (17). MT and its metabolites not only act as direct free radical scavengers but also can upregulate the gene expression of antioxidant enzymes such as catalase, glutathione peroxidase, and superoxide dismutase (18). Besides, melatonin has a wide range of physiological functions, including anti-inflammatory, anti-apoptotic, anti-aging activities immune improvement, sleep promotion, and reproductive modulation (19). In recent years, the effects of melatonin on embryonic development have been extensively studied (20). Melatonin supplementation improved the developmental quality of oocytes and embryos. The effects of melatonin on cryopreservation are also a hot topic of research. A variety of studies have shown that melatonin added to vitrification media improves the quality of vitrification/warming oocytes and their subsequent development by suppressing oxidative stress in human (21, 22), cow (23), and mice (24). Several protective mechanisms of melatonin during cryopreservation have been suggested. However, the majority of these studies are related to gene expression in vitrified embryos after melatonin supplementation and are based on data from microarray (25–27) or bulk RNA-Seq (28, 29) analyses. These analyses provide limited information on the protective mechanisms of melatonin.

In the current study, an advanced single embryo transcriptomics approach will be used to analyze the molecular alterations induced by Cryotop vitrification procedure. This method can determine the process-specific embryonic gene expression alterations during vitrification to confirm the protective role of melatonin. According to the existing multiple ovulation and embryo transfer (MOET) protocol in the lab, melatonin will be added to the Cryotop vitrification culture medium at freeze/warm, and unfrozen sheep morulae. The data obtained in this study will provide new insights into transcriptome alterations induced by vitrification and melatonin and give us a better understanding of the embryo’s development following vitrification and to further improve this process.

## 2. Materials and methods

### 2.1. Chemicals

Unless otherwise indicated, chemicals were purchased from Sigma–Aldrich (St. Louis, MO, United States). The Cryotop devices were purchased from Ingamed (Maringá, Paraná, Brazil). Follicle-Stimulating Hormone (FSH), Pregnant Mare Serum Gonadotropin (PMSG), and Prostaglandin (PG) were purchased from Sansheng biological technology (Ningbo, China); EAZI-BREED-CIDR and Holding medium were purchased from Zoetis (New Jersey, United States).

### 2.2. Experimental design

Vitrified embryo *in vivo* and RNA-Seq: morulae were collected from the uterine horns of 9 Hu sheep and randomly divided into three groups, i.e., 11 in the fresh group (no vitrification), nine in the frozen group (vitrification), and seven in the melatonin group (melatonin, 10^−5^ M melatonin added to the vitrification and warming solution). Morulae were vitrificated and warmed, and the warmed embryos were transferred to Holding solution and recovered at 38.5°C, 5% CO_2_ incubator for 0.5 h. The single embryos were collected for genome amplification and sequenced to analyze differentially expressed genes.

Vitrified embryo *in vitro* and qPCR: sheep ovaries were taken from the slaughter house, COCs were collected, matured *in vitro*, and morulae were collected on day 6 after *in vitro* fertilization, randomly divided into three groups, refer to the previous paragraph for grouping and embryo handling. The single embryos collected were subjected to RT-qPCR after genomic amplification to verify the expression of differential genes analyzed by *in vivo* embryo RNA-Seq data.

### 2.3. Synchronization of superovulation and embryo collection

Ewes aged 2.5–3 years old were chosen as the donors, CIDR + FSH + PMSG procedure was used for superovulation (30). The ewes were treated by inserting a CIDR (progesterone content: 0.3g/capsule; Pharmacia & Upjohn Pty Ltd., Rydalmere, NSW, Australia) into vagina and with intramuscular injection of vitamin AD 3–5 mL at the same time. Ten days after CIDR insertion, FSH (Ovagen; Immuno-Chemical Products Ltd., Auckland, New Zealand) was injected (300–500 IU per donor, based on body weight and operation times to adjust the doses) every 12 h. After the seventh injection, CIDR was removed. PMSG was injected 12 h after CIDR was removed. PMSG was injected at a dose of 330–450 IU per donor. Endoscopic artificial insemination was performed in the donor ewes 48–54 h after CIDR retrieval. 5–6 days after insemination, embryos were recovered by surgical flushing from the uterine horns. Embryos were collected under a stereomicroscope. The status, color, size, number, fullness, and developmental stages were recorded. The embryos were collected surgically by laparotomy under general anesthesia on the sixth day after the first mating. Each uterine horn was flushed with 20 mL pre-warmed phosphate-buffered saline (PBS) containing 3 mg/mL bovine serum albumin (BSA). The embryos were immediately placed in holding solution (Immuno-Chemical Products Ltd.). The morphology of the embryos was assessed and good morulae were used for freezing. The samples were processed in groups referring to the protocol in section 3.

### 2.4. *In vitro* embryo collection

Sheep ovaries were collected from a local slaughterhouse within 3 h, immersed in 25–30°C sterile saline containing penicillin (100 IU/mL) and streptomycin (100 IU/mL), and immediately transported to the laboratory. COCs were collected by cutting ovarian follicles with a razor blade in a 90 mm diameter culture dish. COCs with homogeneous cytoplasm and intact cumulus were selected for the study. The tissue culture medium 199 (TCM-199) supplemented with sodium pyruvate (2.5 mM), L-glutamine (1.0 mM), penicillin (100 IU/mL), streptomycin (100 IU/mL), 15% fetal bovine serum, and cysteamine (0.1 mM) was used as the basal medium. The basal medium added 100 ng/mL EGF, 10 μg/mL FSH, 10 μg/mL LH, and 1 μg/mL estradiol-17β, was used as maturation solution (IVM). COCs were cultured with IVM medium at 38.5°C with 5% CO_2_ for 24 h. Warmed ram’s frozen semen was used for IVF. Then, sperm cells and the mature oocytes were incubated together in synthetic oviduct fluid (SOF) for 18 h at 38.5°C, 5% CO_2_. The SOF supplemented with 1 mM glutamine, 8 mg/mL fatty acid-free BSA, 1% (v/v) Basel Medium Eagle (BME)–essential amino acids, and 1% (v/v) Minimum Essential Medium (MEM)–non-essential amino acids were used as embryo development solution. Then, the zygotes were cultured in embryo development solution at 38.5°C with 5% CO_2_, 5% O_2_, and 90% N_2_. After 6 days of incubation, morulae were processed in groups referring to the protocol in section 3. Individual embryos were transferred to cell lysis buffer under a stereomicroscope. Subsequently, reverse transcription was performed to synthesize the second strand cDNA, its amplification was carried out by 16 PCR cycles with 3’P2 and IS primers.

### 2.5. The processes of vitrification and warming of embryos

Vitrification solutions: pretreatment solution contained 10% ethylene glycol (EG) in DPBS medium, and vitrification solution (EFS35) contained 35% EG (v/v), 30% Ficoll (w/v), and 0.5 M sucrose in DPBS medium. Embryos were incubated in 10% ES medium for 30 s at room temperature and transferred to EFS35 solution for 25 s. The embryos were then transferred to the straws of Cryotop system (Kitazato BioPharma Co. Ltd., Shizuoka, Japan) with a minimal volume of freezing liquid which only wrapped around the embryos. The embryo containing Cryotop was immediately submerged in liquid nitrogen. For embryo warming, the cryopreserved Cryotop straws were immediately immersed in 0.5 M sucrose solution at 37°C. After all, embryos were detached from the Cryotop straws. The embryos were placed in sucrose solution for 5 min, washed in Holding solution three times, and then cultured in 5% CO_2_ for half an hour for recovery. Only embryos of good quality were used for the study.

### 2.6. The single embryo transcriptome sequencing

Single-cell RNA-seq libraries were prepared using an experimental protocol based on single-cell transcriptomics sequencing technology. The *in vivo* samples were treated with acid Tyrode’s solution to remove zona pellucida. The morulae were transferred into 2 μL of lysate buffer under a microscope by pipetting. Reverse transcription was performed using PCR to synthesize second-strand cDNA. The 3′ P2 primers and IS primers were used to amplify the product with 16 PCR cycles. Subsequently, the 96 different barcode labeled single embryo PCR pre-amplification products were pooled and purified using AMPure XP magnetic beads. PCR amplification was then performed with 40 ng of DNA and IS primers. The amplified cDNA was broken into approximately 300 bp fragments using Covaris S2. DNA Fragments were then enriched by incubation with streptavidin C1 magnetic beads (Thermo Fisher) for 1 h. Finally, libraries were constructed using the KAPA Hyper Pre Kit (KAPA Biosystems). All single-cell RNA-seq data were generated on the Illumina HiSeq4000 platform for 150 bp paired-end reads.

### 2.7. Single embryo RNA-Seq data analysis

Based on the original barcode tags for each individual *in vivo* morula, the R2-end sequencing data were split. These R2-end split data were used to classify the R1-end data. The R1-end data were processed with the addition of UMI information and the removal of polyA TSO sequences, and then the mRNA sequences were compared to the sheep genome (oviAri4) from the UCSC database as the reference sequence using HISAT2 (31). The resulting bam files from the comparison were analyzed for transcript counting by using HTSeq software (32). The primary quality control screening criteria were expressed genes with a count greater than 500, total gene expression with a count greater than 1,000, and an alignment rate greater than 20%. The primary qualified data were screened again by selecting the data which had <0.4 mitochondrial expressed genes. For the final quality control sequencing results, the PCA and tSNE downscaling and clustering of cell types were carried out with the Seurat package (33) in R to identify differentially expressed genes (DEGs). *p* < 0.05 was considered statistically significant. Gene ontology (GO) and Kyoto Encyclopedia of Genes and Genomes (KEGG) enrichment analysis were performed based on the differentially expressed genes. To obtain information on the biological functions of over-representation, the Metascape tool^1^ was used (34). GSEA analysis was performed by using the R package clusterProfiler (35). Virtual Inference of protein activity by Enriched Regulon analysis (VIPER) was used to infer regulator activity. The ARACNE algorithm was conducted to construct TRNs and identify some MRs with DEGs from comparisons (36, 37). Regulators with *p* < 0.05 were inferred as driver factors in every two consecutive stages.

### 2.8. RT-qPCR

RT-qPCR was implemented with the ChamQ Universal SYBR qPCR Master Mix (Vazyme, China) by using the CFX96TM Real-Time system (Bio-Rad, United States). Samples were obtained from the *in vitro* embryos described in section 2.4. The primer pairs included HIF1A, 5′-GACCCTGCACTCAACCAAGA-3′ (forward), 5′-TGGGACTGTTAGGCTCAGGT-3′ (reverse); PARP1, 5′-CGTGGACATCGTCAAAGGG-3′ (forward), 5′-TGTTACTACCAATCACCGTGCC-3′ (reverse); PRKDC, 5′-TCTACACGGTCGCCACTCATT-3′ (forward), 5′-TTTCTGACTCTTTGGACCTACGC-3′ (reverse); BTG2, 5′-CTGGAGGAGAACTGGCTGTC-3′ (forward), 5′-AAAACAATGCCCAAGGTCTG-3′ (reverse); ACTB, 5′-GTCACCAACTGGGACGACAT-3′ (forward), 5′-CATCTTCTCACGGTTGGCCT-3′ (reverse); and β-Actin expression was used as a housekeeping gene to normalize the data. The relative expression was quantified using the2^−ΔΔCt^ method and data were expressed using the mean ± SEM.

### 2.9. Data analysis

All the data were analyzed by one-way ANOVA by Tukey *post hoc* comparisons to determine the differences. The software of GraphPad Prism 7.0 was used. *p* < 0.05 was considered to be statistically significant.

## 3. Results

### 3.1. Transcriptome profile of vitrified sheep morula

**Figure 1:**
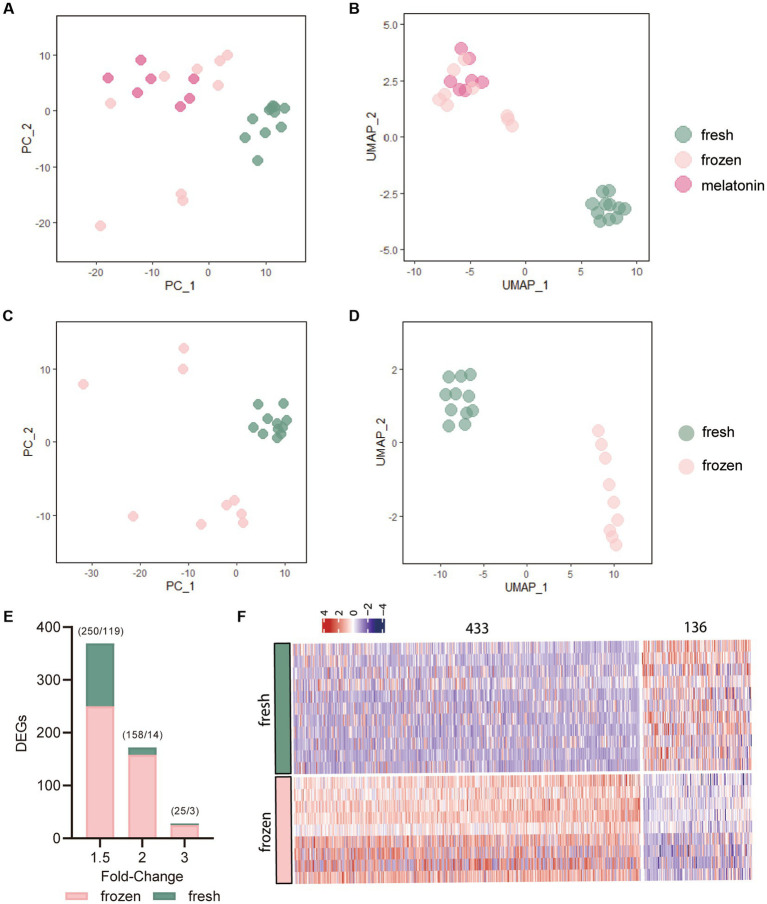
The effects of vitrification on gene expression of morula. **(A,B)** Principal Component Analysis (PCA) and Uniform Manifold Approximation and Projection (UMAP) plots of the expression levels of significantly differentiated 800 genes. Each dot represents a single morula sample. The colors denote the fresh group (green), frozen group (pink), and frozen plus melatonin group (fushcia). **(C,D)** PCA and UMAP plots of the 800 genes in fresh and frozen morula samples based on the most variable expression levels in the dataset. **(E)** Number of differentially expressed genes (DEGs) in vitrified-warmed morulae compared to the fresh control embryos. DEGs were identified from transcriptome analysis using different fold-change values (1.5, 2, and 3) and *p* value of 0.05. Numbers in parentheses indicate the number of up- and downregulated DEGs. **(F)** Heat map of all differentially expressed genes (DEGs) in vitrification-frozen vs. fresh controls. Columns indicate DEGs(*p* < 0.05) and rows indicate vitrification-frozen or control embryo samples. The color keys from blue to red indicate the relative expression of genes from low to high, respectively.

Through data processing and normalization, a total 27 qualified cells were used for further analysis. The normalized read count data from multidimensional scaling plots Principal Component Analysis (PCA) and Uniform Manifold Approximation and Projection (UMAP)， revealed the spatially distributed mappings of the fresh, frozen, and frozen plus melatonin cells (Figures 1A–D). PC_1 and UMAP_1 showed the clear separation of the frozen and fresh cells, however, these separations were not obvious between the frozen and frozen plus melatonin cells. A restriction threshold of differences was a value of *p* < 0.05 and cutoff criteria for DEGs were changed in the expression < −1.5 and > 1.5-fold. Total 569 DEGs were identified in the frozen group compared to the fresh group, and Figure 1E revealed the distribution of DEGs at different fold-change values. More specifically, as shown in the heat map (Figure 1F), 433 genes were upregulated (Supplementary Table S1), while 136 were downregulated (Supplementary Table S2).

**Figure 2:**
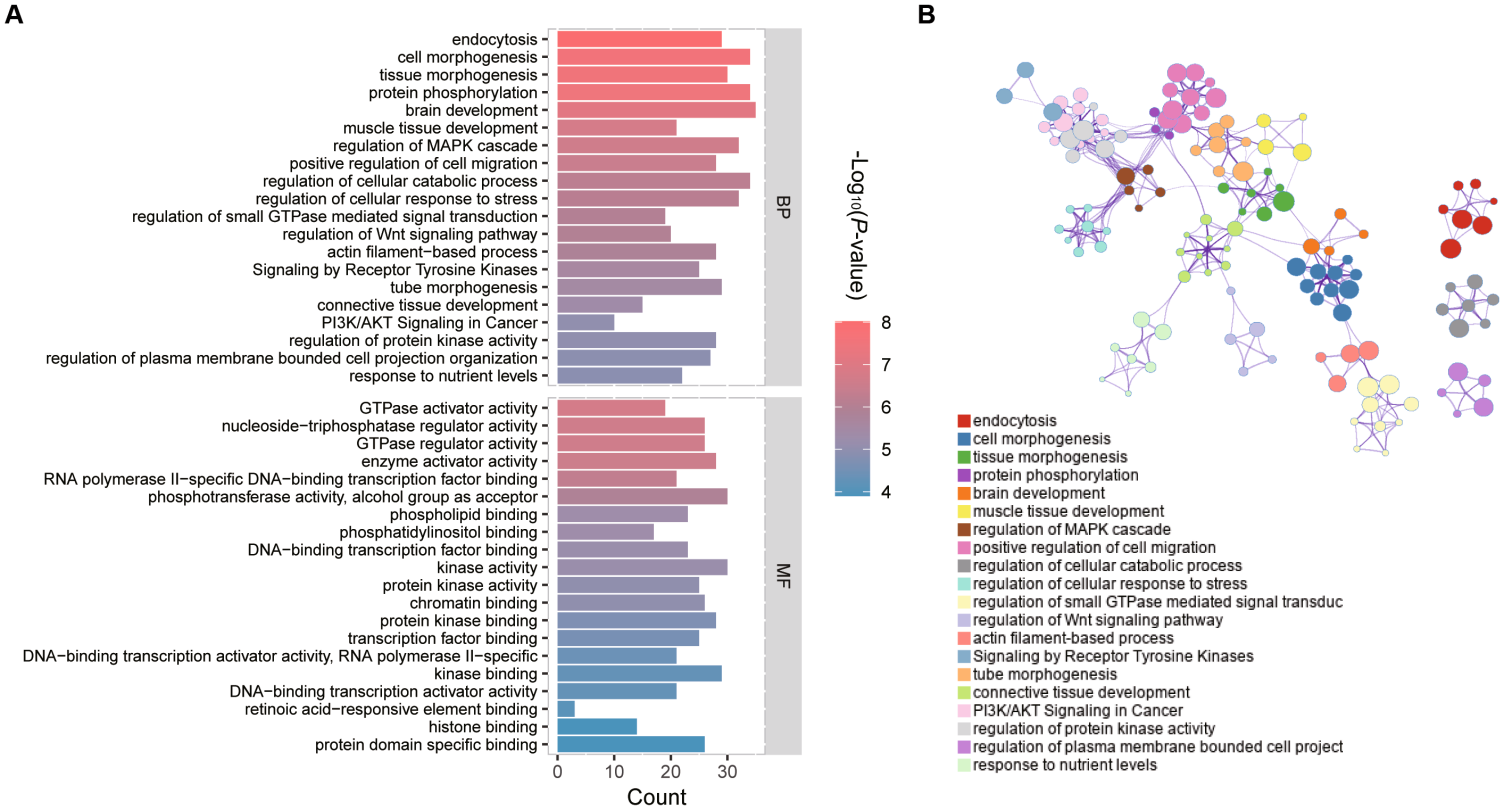
GO functional enrichment analysis of differentially expressed genes (DEGs) in vitrification vs. fresh morula. **(A)** GO analysis of differentially expressed genes (DEGs); horizontal coordinates indicate the number of DEGs in each entry. **(B)** Network of enriched terms colored by cluster identity, where nodes that share the same cluster identity are typically close to each other.

The R and the tool Metscape were used to identify the alterations of the embryonic transcriptome caused by vitrification. The terms and pathways of expressions between vitrification and fresh intergroup comparisons were analyzed by GO through the functional annotation database in the Metascape tool (Supplementary Table S3). Figure 2A showed the top 20 clusters of GO terms biological process (BP) and molecular function (MF). Genes altered in vitrification vs. fresh group were associated with regulations of typical Wnt signaling, MAPK signaling, PI3K/AKT signaling and protein kinase pathways. The regulatory network of the BP process depicted by Cytoscape was illustrated in Figure 2B.

**Figure 3:**
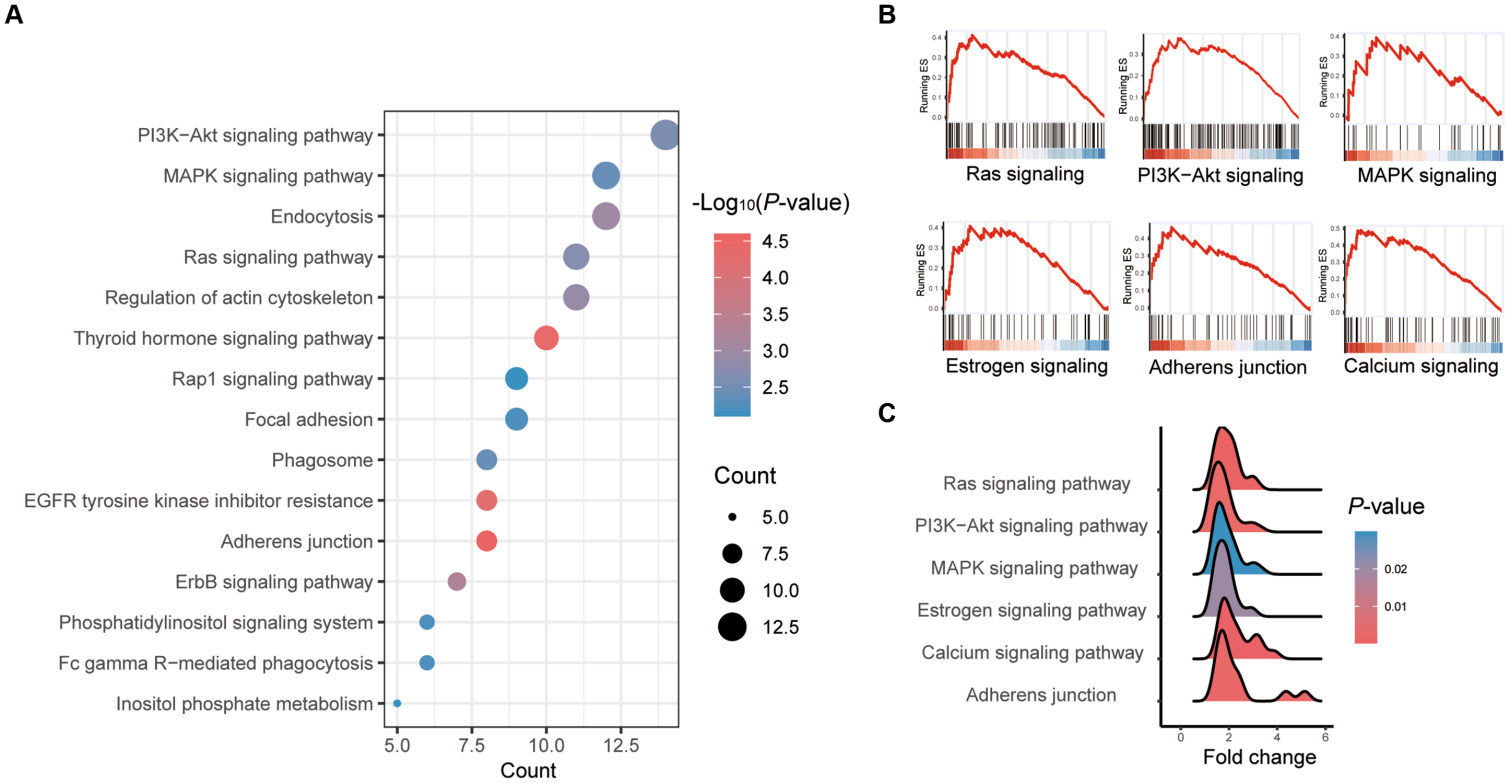
Pathway enrichment analysis of differentially expressed genes (DEGs) in vitrified frozen vs. fresh morula. **(A)** Results from KEGG analysis; the color of each dot indicates the *p* value of the two sets of differentially expressed genes (DEGs), and the size of the dot indicates the number of DEGs in each KEGG pathway. **(B)** The results from GSEA analysis. **(C)** Fold of all genes in the GSEA pathway that are highly expressed in the vitrification morula.

The most enriched KEGG pathway among all significantly differential genes between vitrification and fresh morula was the “adhesion process,” and the most enriched pathway was the “PI3K-Akt signaling pathway” (Supplementary Table S4; Figure 3A). The overall changes in gene set during the process of vitrification analyzed by GSEA were shown in Figure 3B. Vitrification significantly promoted Ras, PI3K-Akt, MAPK, estrogen, and calcium signaling pathways, as well as the adhesion process. Genes of these pathways were significantly upregulated after vitrification (Figure 3C).

### 3.2. The effects of melatonin on transcriptome of morula vitrification

**Figure 4:**
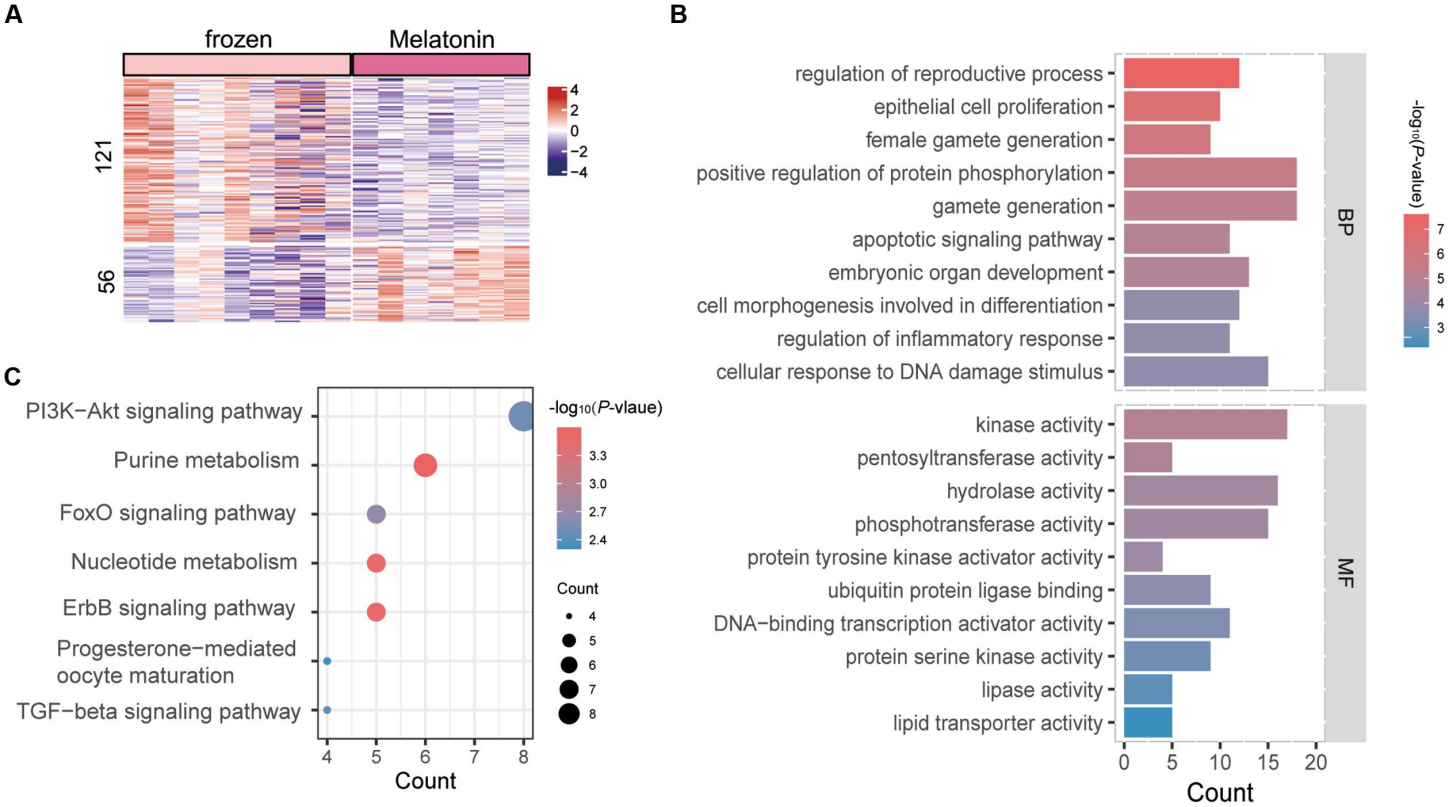
Enrichment analysis of differentially expressed genes (DEGs) in vitrification vs. melatonin plus vitrification morula. **(A)** Heat map of all differentially expressed genes (DEGs). Rows indicate DEGs (*p* < 0.05) and columns indicate vitrification or melatonin plus vitrification embryo samples. The color keys from blue to red indicate the relative expression of genes from low to high. **(B)** GO analysis; horizontal coordinates indicate the number of DEGs in each entry. **(C)** KEGG analysis; the color of each dot indicates the *p* value of the two sets of differentially expressed genes (DEGs), and the size of the dot indicates the number of DEGs in each KEGG pathway.

The effect of melatonin on the transcriptional profile of *in vivo*-derived sheep morula was analyzed during vitrification (frozen vs. frozen plus melatonin). The restriction threshold for the analysis was set at a *p* value of 0.05 and various ploidy change cutoffs. A list of DEGs criteria for ploidy changes <−1 and > 1 was used for subsequent analysis. A total of 177 DEGs were identified between them (Supplementary Table S5), of which 121 genes were upregulated in vitrification group, while 56 were downregulated (Figure 4A). GO analysis was performed for all DEGs, and Figure 4B demonstrated the top 10 clusters for BP and MF (Supplementary Table S6). Genes altered between the two groups in BP which were associated with regulation of reproductive development, gamete production, regulation of protein phosphorylation, apoptotic signaling pathways, and cellular, responsed to DNA damage stimuli. Entries enriched in the MF and KEGG pathways which included those related to the vitrification process in the kinase activity pathway, ErbB signaling pathway, and PI3K-Akt signaling pathway (Figures 4B,C).

### 3.3. The alterations of key transcription factors during morula vitrification and the effects of melatonin on them

**Figure 5:**
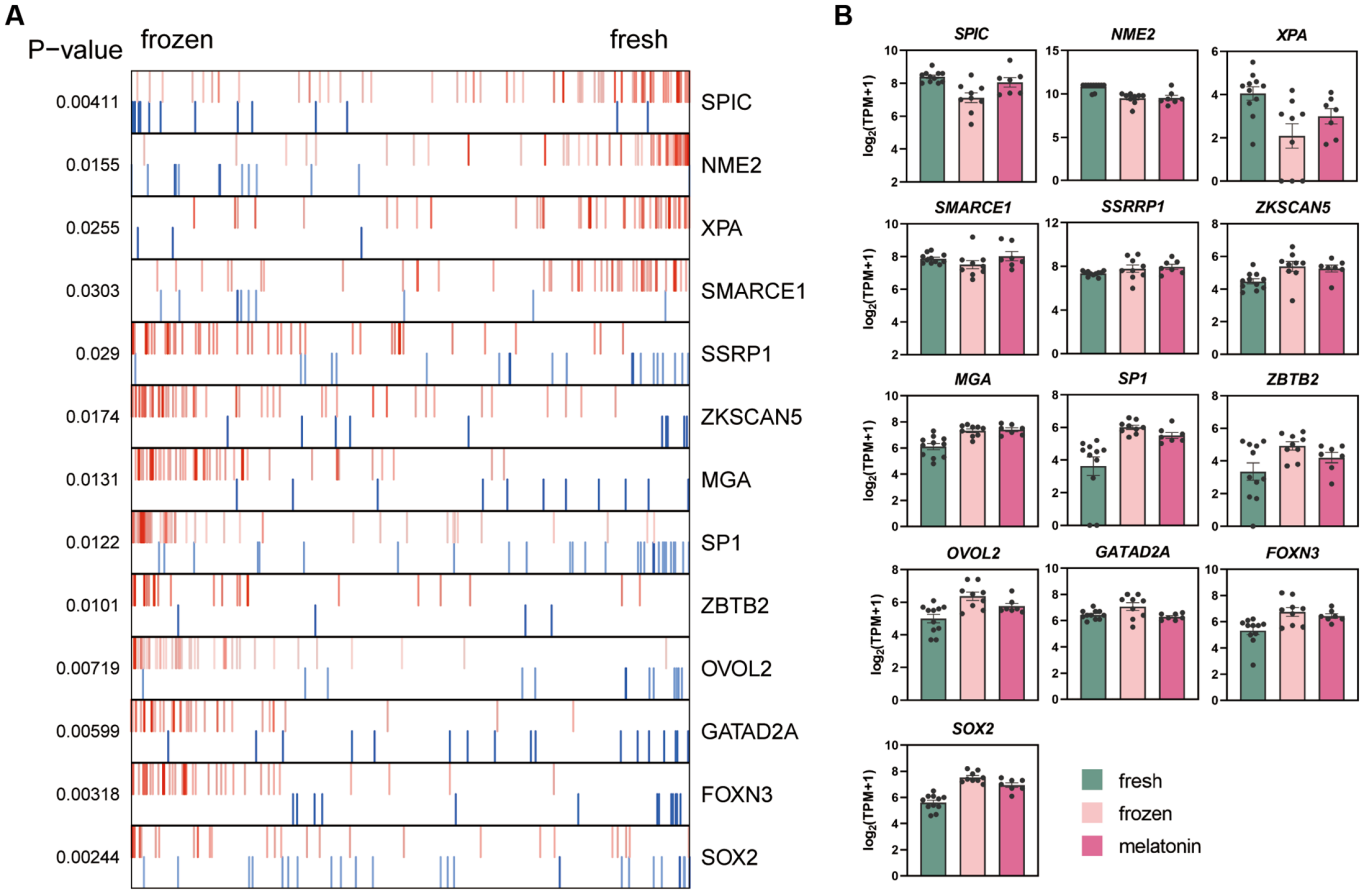
Effects of vitrification and melatonin plus vitrification of morula on the key gene regulators (TFs) analyzed with VIPER. **(A)** Plot produced by VIPER presents the top 13 upregulated and downregulated TFs. **(B)** The bar graph shows the relative expression levels of the genes [log2 (TPM + 1)]. The colors denote the fresh group (green), frozen group (pink), and melatonin group (fushcia).

The ARACNe and VIPER algorithms which were used to identify the key regulators altered during the vitrification process. These enrichment plots of TF master regulators which identified at *p* < 0.05 in analysis were shown in Figure 5A. The activities of SPIC, NME2, XPA, and SMARCE1 were inhibited in vitrifying embryos, and SSRP1, ZKSCAN5, MGA, SP1, ZBTB2, OVOL2, GATAD2A, FOXN3, and SOX2 were activated. The relative expression levels of TF master regulators in the three experimental groups were shown in Figure 5B.

### 3.4. Validation of RNA-Seq results

**Figure 6:**
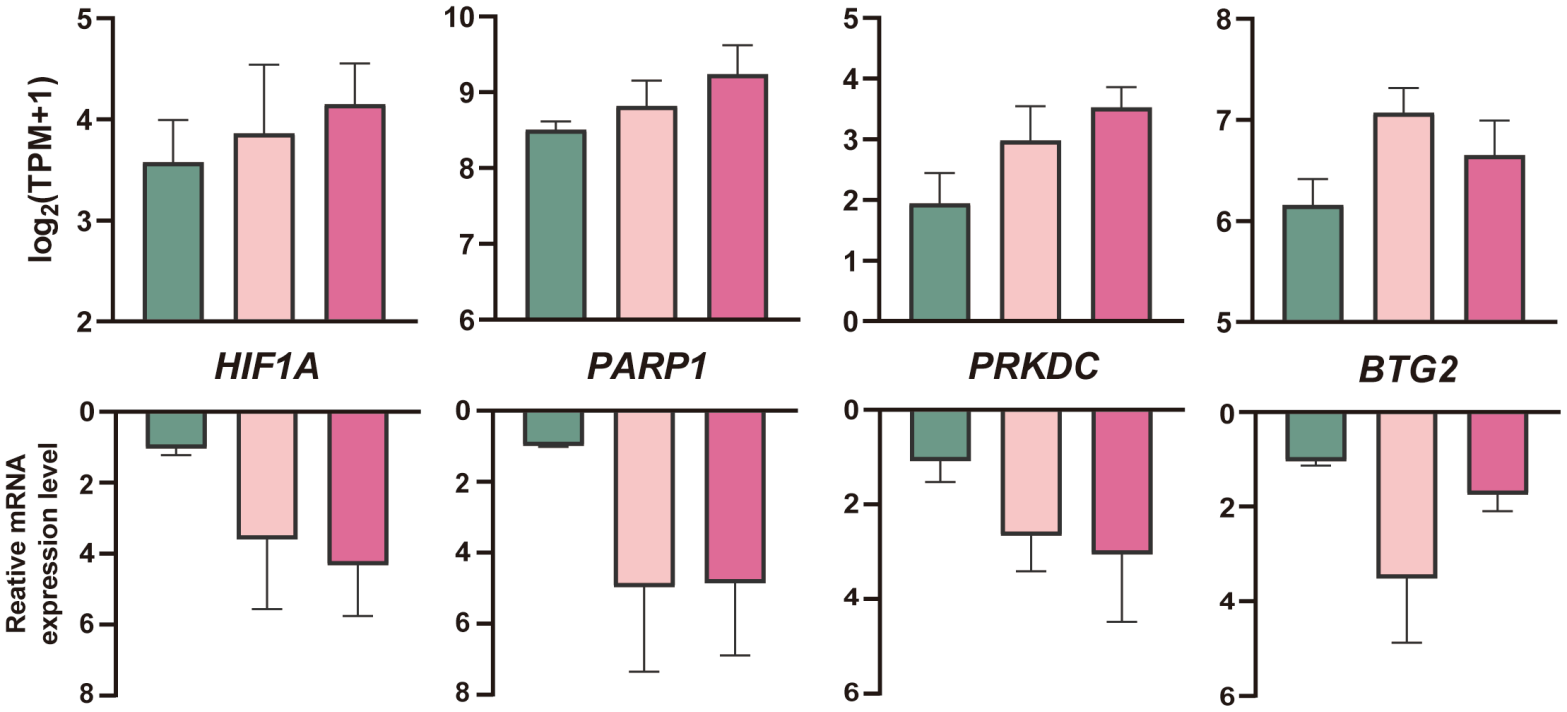
Quantification (mean ± SEM) of mRNA profiles of four genes in *in vitro* produced sheep blastocysts after cryopreservation with RNA-Seq analyses (up) and qRT-PCR (down, *n* = 3 replicates per morula group). The colors denote the fresh (green), vitrification, and melatonin plus vitrification (fushcia).

Embryos generated *in vitro* were grouped and vitrified in the same way as *in vivo* embryos. qPCR results of *in vitro* embryos were compared with RNA-Seq results of *in vivo* embryos. Based on RNA-Seq data and function analysis, four genes (HIF1A, PARP1, PRKDC, and BTG2) of *in vitro* cultured morulae processed with vitrification were selected for validation by qRT-PCR. The compression RNA-Seq and qRT-PCR data among vitrification, melatonin plus vitrification, and *in vivo* control morula were summarized in Figure 6.

## 4. Discussion

In this study, we have provided the transcriptional profiles of sheep morulae after which the vitrification procedure was performed. The data from RNA-sequencing revealed several important transcriptional alterations, which may reflect the molecular damage caused by the frozen temperature and indicate the molecular healing process of the embryos to overcome the impact of vitrification. In recent years, evidence has shown that melatonin acts as an antioxidant to enhance the developmental capacity of cryopreserved embryos. Therefore, in this study, we also investigated the effects of melatonin on vitrification associated transcriptome alterations in sheep morula.

Currently, microarrays (38) and bulk RNA-Seq are widely used to study the influences of vitrification on embryos. In this study, these methods were used to analyze the transcriptome profile of the vitrified sheep embryos at the single embryo level. The sequencing results of single embryos can detect more details on the changes in gene expression caused by vitrification and also have more chances to identify new differential genes. Our results confirmed a previous report that the majority of DEGs are upregulated during the process of vitrification (29). However, we found the effect of vitrification on the number of DEGs to be minor. In terms of expression levels, only 27 DEGs in vitrified morulae showed changes greater than 3-fold. The within-group variation in vitrified morulae was greater than that in other treatments, which may imply that different embryos respond differently to freezing. Meanwhile, the PCAs of vitrification and melatonin plus vitrification groups were clustered into one group, indicating that the addition of melatonin had less effect on the transcriptional expression of vitrified morulae.

Gene ontology and KEGG enrichment analysis of vitrified morulae showed that GO terms which involved in GO metabolism, tissue development, cell signaling biological processes, and DEGs associated with kinase activity and transcription factor binding activity were significantly enriched. The results indicate that the vitrification/warming processes induce responses that are mainly related to cellular metabolism in morulae. During the process of vitrification, embryos turn into glass-like solid phase to avoid formation of intracellular ice and ice crystal mechanical damage, while at the same time, vitrification causes irreversible damage to the cell membrane of embryo (39). This is consistent with our findings, in which the important biological processes closely related to the function of the cell membrane (endocytosis and adhesive junctions) were significantly enriched. In both domestic animals and humans, adhesive interactions between adjacent cells are essential for embryogenesis as well as for tissue morphogenesis and renewal (40). Endocytosis is essential in early embryonic development (41). In mice embryos, clathrin-mediated endocytosis plays a key role in the progression of early embryogenesis and selective degradation of oocyte-derived plasma membrane proteins (42). The alterations of genes in these two functional pathways could significantly impact the development of vitrified morulae. Our results showed that the genes in these two functional pathways were upregulated, indicating their importance for vitrified morulae survival.

Among the pathways obtained from vitrified morulae DEGs enrichment, the signaling pathway (PI3K/AKT, MAPK, and Ras) entries were significantly enriched for their regulatory involvement in embryonic development, oxidative stress. The phosphoinositide 3-kinase/protein kinase B (PI3K/AKT) and mitogen-activated protein kinase (MAPK) pathways play critical roles in regulating a variety of cellular functions, including proliferation, survival, growth, transcription, and protein synthesis (43). The MAPK pathway is involved in stress responses triggered by many extracellular and intracellular stimuli, and a significant enrichment of the MAPK pathway was also observed in vitrified porcine blastocysts (27). the PI3K/AKT pathway is an important signal that regulates apoptosis by modulating oxidative stress. It was found that this pathway was involved in the response to oxidative stress during vitrification/warming process and the developmental potential of the warming mouse oocytes (44).

Melatonin as a potent antioxidant has been widely used to enhance the developmental competence of cryopreserved animal oocytes (24). Our results showed that the TGFβ and FoxO pathways were significantly enriched in the morulae of melatonin plus vitrification treatment compared to those of vitrification alone. These pathways regulate the pluripotency and pregnancy of stem cells involved in embryonic development. Enrichment of the signaling pathway has also been reported in vitrified porcine COCs (45, 46), as well as in vitrified mouse blastocysts, indicated by increased level of miRNA that was associated with TGFβ upregulation (47). The FoxO pathway is also activated under stress conditions (48); FoxO molecules have been found in the inner cell mass of mouse blastocysts (49) to upregulate target genes, thereby promoting cell cycle stalled genes and increasing resistance to stress.

Our study revealed that some apoptosis-related genes that are highly expressed after vitrification were repressed, while factors that promote DNA damage repair were upregulated with melatonin treatment. Several altered transcription factors during vitrification were identified by ARACNe method. A significant decrease in XPA activity after vitrification was observed. and melatonin alleviates this decrease in XPA expression caused by vitrification. XPA is a major subunit of the Nucleotide excision repair (NER) system, which efficiently recognizes DNA damage and then recruits NER proteins to the damaged region (50, 51). NER is a versatile repair pathway and is capable of removing a broad spectrum of DNA helix damage (52). XPA is a key protein in the NER and is important for DNA damage validation and recruitment of other NER proteins (53). Many studies have shown that the lack of XPA leads to complete failure of the NER (54).

In addition, XPA is a direct target of hypoxia-inducible factor 1 alpha (HIF-1α), and binding of HIF-1α to HRE in the XPA promoter region significantly upregulates XPA expression (55). We found melatonin also upregulated HIF1A expression, and this may be one of the mechanisms by which melatonin indirectly acts on the XPA to suppress vitrification damage. XPA can interact with PARP1, which facilitated by the Poly-ADP-ribosylation (PARylation) of XPA. The direct interaction of XPA-PARP1 further stimulates PARP1 activity and promotes additional Poly-ADP-ribosylation events and chromatin structure opening (56). PARP1 was also significantly upregulated in melatonin plus vitrification compared to the vitrification alone group. The circadian rhythm could also influence NER by directly modifying XPA levels (57). In mice brain, liver, and skin, XPA and NER exhibit strong circadian rhythmicity (58). Melatonin is a potent circadian rhythm regulator; therefore, it may regulate the XPA via the circadian mechanism. Further studies on the regulatory relationship between the two would be of interest.

## 5. Conclusion

Our findings revealed specific alterations in embryonic gene expression caused by the vitrification process and identified the potential pathways, which involved in vitrification caused damage on morulae. These alterations caused by vitrification were mainly enriched in adhesion processes, endocytosis, and kinase activity. In addition, we uncovered genes that are involved in the protective role of melatonin on vitrification damage. These genes include XPA and HIF1A. To the best of our knowledge, this is the first report on the alterations of transcriptome profile caused by vitrification/warming process on the *in vivo*-derived sheep morulae at the single embryo level. This study contributes to the understanding of the consequences of vitrification/warming procedures on embryo quality and developmental competence, not only in sheep, but also in other mammalian species. In addition, the protective mechanisms of melatonin in vitrification against morulae damage were also explored. These observations provide an important molecular mechanism for further optimizing the cryopreservation of embryos and other cells.

## Data availability statement

The datasets presented in this study can be found in online repositories. The names of the repository/repositories and accession number(s) can be found below: https://www.ncbi.nlm.nih.gov/; PRJNA971313.

## Ethics statement

The animal study was reviewed and approved by China Agricultural University Laboratory Animal Welfare and Animal Experimental Ethical Inspection Committee.

## Author contributions

PJ: conceptualization. GL: funding acquisition. PJ, LY, YJ, DL, YY, WM, and DY: investigation. FL, AW, and KY: project administration and resources. LZ and GL: supervision. YL and MZ: visualization. YL: writing–original draft. LZ, SG, and GL: writing–review and editing. All authors contributed to the article and approved the submitted version.

## Funding

This research was funded by the Major Science and Technology Project of Inner Mongolia (2021ZD0023-1), Biological Breeding Project-Topic4 (2022ZD0401404), and National Key Research and development Program (2021YFD1300903).

## Conflict of interest

FL is employed by Inner Mongolia Golden Grassland Ecological Technology Group Co., Ltd.

The remaining authors declare that the research was conducted in the absence of any commercial or financial relationships that could be construed as a potential conflict of interest.

## Publisher’s note

All claims expressed in this article are solely those of the authors and do not necessarily represent those of their affiliated organizations, or those of the publisher, the editors and the reviewers. Any product that may be evaluated in this article, or claim that may be made by its manufacturer, is not guaranteed or endorsed by the publisher.

## Footnotes

^1^http://metascape.org

## Supplementary material

The Supplementary material for this article can be found at: https://www.frontiersin.org/articles/10.3389/fvets.2023.1212047/full#supplementary-material
